# Updated Genome Sequence and Annotation for the Full Genome of Pseudomonas protegens CHA0

**DOI:** 10.1128/MRA.01002-19

**Published:** 2019-09-26

**Authors:** Theo H. M. Smits, Fabio Rezzonico, David Frasson, Pilar Vesga, Jordan Vacheron, Jochen Blom, Joël F. Pothier, Christoph Keel, Monika Maurhofer, Martin Sievers

**Affiliations:** a Environmental Genomics and Systems Biology Research Group, Institute of Natural Resource Sciences, Zurich University of Applied Sciences (ZHAW), Wädenswil, Switzerland; b Microbiology and Molecular Biology Research Group, Institute of Chemistry and Biotechnology, Zurich University of Applied Sciences (ZHAW), Wädenswil, Switzerland; c ETH Zür, Plant Pathology, Institute of Integrative Biology, Zurich, Switzerland; d Department of Fundamental Microbiology, Quartier UNIL-Sorge, University of Lausanne, Lausanne, Switzerland; e Bioinformatics and Systems Biology, Justus-Liebig-Universität, Giessen, Germany; Indiana University, Bloomington

## Abstract

Minor differences in the previously obtained genome of Pseudomonas protegens CHA0 were detected after resequencing the strain. Based on this, the genome size slightly increased. Additionally, we performed a manual annotation of genes involved in biocontrol and insect pathogenicity. This annotation version will be the basis for upcoming genome studies.

## ANNOUNCEMENT

Pseudomonas protegens CHA0 has a long history as a biocontrol agent. Many features and the regulation thereof have been described for this strain that are involved in the process of biocontrol against fungal and oomycete plant pathogens (1–3). Additionally, the organism has been recognized as an effective insect pathogen that can kill larvae of different plant pest insects after oral infection by the bacterium (4, 5). In addition, several features contributing to insect pathogenicity were described already (2, 4, 6). While the genome sequence of this strain was published a few years ago (7), the pathogen control and insect pathogenicity determinants were only poorly included.

We have resequenced the genome of P. protegens CHA0 starting with genomic DNA from its accession number CCOS 2 at the Culture Collection of Switzerland (CCOS). The strain was grown in LB broth at 28°C for 1 day. Total DNA was extracted from the pure culture using the DNeasy blood and tissue kit (Qiagen, Hilden, Germany). Genomic library preparation and genome sequencing were outsourced to GATC Biotech, AG (Constance, Germany). Libraries were prepared using a SPRIworks fragment library system I (Beckman Coulter, Brea, CA), following the manufacturer’s instructions. The TruSeq paired-end (PE) cluster kit v3-cBot-HS (Illumina, San Diego, CA) was used for cluster generation. Sequencing was performed on a HiSeq 2000 Illumina sequencer with 2 × 50-bp paired-end reads using the TruSeq SBS kit v3-HS (Illumina). A total of 97,357,690 quality-filtered reads were obtained from GATC, giving an approximate coverage of 700×. For *de novo* assembly using SeqMan NGen v12.2 (DNAStar, Madison, WI) with standard settings, only 8,500,000 reads (55× coverage) were used. Repeated cycles of read mapping with the SeqMan NGen software and inspection in different subroutines of the Lasergene package (DNAStar) yielded a complete genome of 6,868,156 bp with a G+C content of 63.39%. Based on a small region close to an rRNA region, the genome was 176 bp larger than the previous version (7). Additionally, few indels mainly in homopolymer regions were observed.

Annotation of the genome sequence was done in GenDB (8), with manual improvements. Comparative genomics was done in EDGAR v2.3 (9) using the parameters defined by Smits et al. (10). A comparison of the annotations indicated a difference between the two genome sequences. The two genomes share 5,995 genes, whereas 142 and 120 singletons were observed for our version, respectively, to the previous one, with the majority representing hypothetical proteins. Furthermore, manual annotation of all known biocontrol features and insect pathogenicity factors was performed. This version of the genome is now suited for use in comparative genomics studies to study different aspects of biocontrol and insect pathogenicity.

### Data availability.

The genome sequence of P. protegens CHA0 (CCOS 2) was deposited at DDBJ/EMBL/GenBank under the BioProject number PRJEB28440 and accession number LS999205. The version described in this paper is version LS999205.1. Raw sequence reads (Illumina) have been deposited at the EMBL under accession number ERR3047520.
